# What’s in store for females after breaking the glass ceiling? Evidence from the Chinese audit market

**DOI:** 10.3389/fpsyg.2023.1321391

**Published:** 2023-11-21

**Authors:** Hanxiu Cheng, Jie Wang

**Affiliations:** ^1^School of Economics and Management, Nanjing University of Science and Technology, Nanjing, China; ^2^School of Business, Anhui University of Technology, Maanshan, Anhui, China

**Keywords:** glass ceiling, gender discrimination, female auditor, partner, audit firm

## Abstract

Given that female auditors’ representation in the audit market has averagely caught up to that of males, yet remains insufficient at the partner level, it is imperative to investigate gender discrimination in public accounting firms. Using data from the Chinese audit market, this paper analyzes the glass ceiling phenomenon faced by females as they aspire to promotion to partner positions. It also explores the professional barriers that may impede their career progression post-promotion. The findings illuminate that the opportunities for female promotion to partner positions are notably lower than for males. Furthermore, after their elevation to partner roles, females are more likely to be allocated to clients grappling with financial distress and high-risk situations. In contrast, opportunities to engage with auditing important clients are diminished, particularly within male-dominated audit firms. Additionally, the study reveals that female promotion to partner positions heightens their prospects for assuming the lead auditor role in audit projects. However, this phenomenon predominantly materializes within audit firms characterized by a higher proportion of female auditors. Instead, females face more significant challenges in garnering recognition within male-dominated audit firms. Lastly, the research examines investor reactions to female promotion to partner, revealing a generally negative response. In summary, this study contributes to a comprehensive exploration of gender discrimination within the public accounting firms, shedding light on women’s career development challenges after breaking the glass ceiling.

## 1 Introduction

To enhance the status of females in the labor market and promote gender equality at a societal level, significant efforts have been made globally over the past few decades (Bertrand et al., 2019; UN Women, 2023). However, female representation in leadership roles remains inadequate (Rattan et al., 2019). This phenomenon is particularly pronounced in public accounting firms. The career trajectory of auditors in audit firms has historically been characterized as “up or out,” with promotion to partner being the highest professional pursuit in the auditing industry. Nevertheless, males hold the dominant position in the audit market, and females are underrepresented in partner positions, which has been a long-standing issue. According to the “2017 Registered Accountant Industry Development and Management Report” released by the Accounting Department of the Ministry of Finance of China, in 2017, females comprised 50.33% of Certified Public Accountants (CPAs), nearly achieving gender equality. However, when scrutinizing the proportion of partners, a stark contrast emerges, with females accounting for only 41.96%, a substantial deficit of 16.08 percentage points compared to male partners. Within the 40 audit firms possessing securities qualifications, the female proportion is even lower, standing at a mere 31.08%, lagging behind the industry average by 10.88 percentage points. Similar observations of this gender disparity have been made in other countries (Gammie et al., 2010; Almer et al., 2021), indicating the existence of “vertical gender segregation” within the auditing industry (Hull and Umansky, 1997; Almer et al., 2022). Therefore, despite the appearance of gender equality throughout the auditing industry, this paper posits that there is reason to suspect the presence of a glass ceiling for females in their professional development to partner in the Chinese audit market.

Notably, once females break through the glass ceiling and are promoted to partner, will they face career development dilemmas again? This question involves doubts about female competence as well as potential gender discrimination. The underrepresentation of females at the partner level has long been an unresolved issue. However, when females are promoted to partner, their competence will no longer be a questionable concern. It is reasonable to believe that females promoted to partner positions are comparable to, and perhaps even superior to, males in competence. Using data from the Belgian public accounting profession, Hardies et al. (2021) find that female auditors have to be more productive than males to be promoted to partner. However, females are not valued as much as they should be after being promoted to partner, as evidenced by the fact that female partners are less likely to be assigned to highly reputable clients (Almer et al., 2021). Therefore, examining whether there is a significant difference in the types of clients that females serve after being promoted to partner in the Chinese audit market is worthwhile.

Using hand-collected data on partner promotion in Chinese audit firms, this paper delves into the challenges faced by females promoted to partner in audit firms and their career development after promotion to partner, thus providing new perspectives for academic research on the glass ceiling. Specifically, this study aims to answer four key research questions to shed light on gender inequality in the auditing careers of females. First, this paper explores whether there is a glass ceiling effect for females in the auditing market. This issue is directly related to potential career advancement barriers to promotion due to gender bias. Second, we analyze whether females are hindered in audit practices once they break through the glass ceiling successfully. Specifically, we investigate whether there is a significant difference in client allocation among female partners and whether this difference is more pronounced in male-dominated audit firms. This question contributes to understanding whether gender factors influence the responsibilities and opportunities for females in leadership. Subsequently, the paper focuses on the extent to which audit firms value female partners, i.e., whether or not they will allow female partners to lead more in audit engagement. Finally, it tests how investors react to promoting females to partner in the capital market. By investigating these critical issues, this study aims to provide theoretical and empirical support to promote gender equality and female careers in auditing.

This paper contributes to the literature on female career development and the glass ceiling phenomenon in multiple ways. Firstly, by analyzing the likelihood of female auditors in the Chinese audit market promotion to partners, this study broadens the research on the glass ceiling phenomenon. While prior studies have discussed the glass ceiling phenomenon for women in public accounting firms, many of these studies primarily rely on interviews and surveys (Cohen et al., 2020; Almer et al., 2021). Fewer studies have conducted empirical analyses of the barriers female auditors face in promotion, primarily focusing on countries such as Germany (Downar et al., 2020) and Belgium (Hardies et al., 2021). However, the audit market in China possesses distinctive characteristics, including higher fragmentation and the absence of Big4 dominance. Therefore, conclusions drawn from data in other countries may not necessarily apply to the Chinese audit market. This paper, by manually collecting data in China, empirically tests the obstacles faced by females in promotion to partner, thereby expanding the research on female career development within the audit market and supplementing the literature on the glass ceiling phenomenon.

Secondly, this paper advances research on the potential glass cliff phenomenon females may face upon entering leadership roles by analyzing differences in client allocations following female promotion to partner. The study reveals that after females are promoted to partner roles, they are more likely to be assigned to high-risk clients and less likely to be assigned to essential audit clients, with this difference being particularly pronounced in male-dominated audit firms. By examining how audit firms differentially handle female partners in auditing practices, this paper elucidates potential career development challenges faced by females entering leadership roles and provides empirical evidence from public accounting firms regarding the glass cliff phenomenon.

Lastly, the paper finds that females promoted to partner are often not given sufficient attention within audit firms and are frequently not appointed as lead auditors for audit projects. Additionally, investors’ reactions to female promotion to partner are generally negative. These research findings demonstrate that, even though females have broken the glass ceiling and reached leadership positions, they still fail to gain full recognition within organizations and even in capital markets. Therefore, this study contributes strong evidence to the gender discrimination literature, emphasizing that gender equality remains a significant issue.

This paper is arranged as follows. In Section 2, the paper reviews prior literature and proposes the hypothesis. Section 3 describes the data sample and research design. Section 4 presents the results. Additional tests are performed in Section 5. The conclusions are summarized in Section 6.

## 2 Literature review and hypotheses development

### 2.1 Underrepresentation of females in leadership

In 1986, Hymowitz and Schellhardt (1986) introduced the “glass ceiling” concept to describe invisible barriers that hinder females from advancing to high-level positions within organizations. Since then, a substantial body of literature has emerged, discussing the factors that affect females’ promotion and how they overcome the glass ceiling. Nevertheless, females continue to be underrepresented in top positions in the labor market. Literature on influencing female promotion focuses on the following aspects: (1) Gender bias. Research indicates that gender bias is a significant phenomenon during recruitment and promotion, where employers prefer male candidates over equally qualified female candidates. This gender bias is rooted in societal and cultural norms and stereotypes (Rudman, 1998; Rudman and Glick, 2001), impacting how employers evaluate and make decisions concerning employees of different genders. A prevalent societal belief exists that females should bear the primary responsibility for caregiving in families. This belief might lead employers to question females’ ability to balance work and family responsibilities, particularly in high-level positions. Even if females are promoted to leadership roles, employers may doubt their competence and worry that family demands might hinder their performance. This misconception could result in a preference for male candidates, as they are perceived as more dedicated to their work (Heilman, 1983). Furthermore, males are often considered to possess leadership qualities, a stereotype associated with traditional masculinity traits such as confidence, decisiveness, and competitiveness. Consequently, in leadership selection, males usually have an advantage, while females may be excluded due to a lack of these perceived qualities (Eagly and Karau, 2002; Heilman, 2012). Gender bias presents a significant obstacle to females’ career development, resulting in unfair treatment regarding promotions and compensation (Eagly and Carli, 2007). (2) Gender discrimination. Gender discrimination is a deeply rooted issue in the labor market that continuously affects females’ upward mobility (Hultin and Szulkin, 1999). Although laws prohibiting gender discrimination have been enacted worldwide, the problem persists. Gender discrimination often disadvantages females in competitive professional settings, even when they possess considerable skills and backgrounds (Heilman, 1983; Spurr, 1990). Females with skills, experience, and performance equal to their male counterparts may still be underestimated or overlooked (Powell and Butterfield, 1994; Hultin and Szulkin, 1999). This phenomenon is commonly referred to as the “glass ceiling,” implying that females seem to face subtle barriers to career development (Morrison et al., 1987; Powell and Butterfield, 2015). Gender discrimination becomes especially apparent in male-dominated work environments (Dalton et al., 2014). Nonetheless, research has shown that increasing the representation of women in management roles appears to reduce the gender wage gap (Cohen and Huffman, 2007) and alleviate the obstacles to females’ promotion (Gorman, 2005; Kurtulus and Tomaskovic-Devey, 2012). (3) Self-selection. Psychological studies suggest that females tend to avoid competition, while males are more willing to embrace it (Niederle and Vesterlund, 2011). Females often actively avoid high-level management positions that require competitive incentives (Gneezy et al., 2003; Niederle and Vesterlund, 2007, 2011). This avoidance behavior may stem from various factors, including aversion to risk and concerns about work-related stress (Barker and Monks, 1998). For instance, females may prioritize balancing their professional and family lives. Influenced by societal and cultural norms, females often shoulder more family responsibilities, leading to concerns about the stress and time demands associated with leadership positions (Heilman, 1983). This perspective may make female less willing to embrace competitive positions, as they fear the potential impact on their roles within their families. Additionally, Gneezy et al. (2003) suggest that in non-competitive environments, women and men perform equally, but as the level of competition increases, women’s performance gradually falls behind men’s. This further emphasizes that females may be more likely to withdraw or behave conservatively in competitive environments. This trend may explain why females are underrepresented in top positions, partly due to their own choices. Niederle and Vesterlund (2007) conduct experimental research to identify the reasons for females’ underrepresentation in high-level leadership positions and sought explanations beyond gender discrimination. Their research results further confirm that females tend to act conservatively in competitive environments, highlighting the impact of gender differences on career choices and promotions in the leadership positions.

### 2.2 Female promotion to partner and the glass ceiling effect

Becoming a partner in the audit firm is considered a sign of success in auditing (Davidson and Dalby, 1993). Similar to shareholders in a corporation, partners own shares of the audit firm and share in the residual profits. However, in contrast, partners not only hold ownership stakes in the audit firm but also actively engage in the execution of auditing practices. They play a crucial role in the audit firm’s operation and client management, actively engaging in audit projects. Nevertheless, males have been dominant in professional service firms like audit firms. Following the principle of homophily, male partners often tend to favor individuals with similar backgrounds when selecting new partners (Dalton et al., 2014). Consequently, this gender disparity is particularly pronounced at the partner level. Numerous studies indicate that female promotion to partner faces challenges (Ragins et al., 1998; Almer et al., 2012; Cohen et al., 2020). While the proportion of female auditors has been gradually increasing throughout the auditing market and has reached parity with male counterparts, female representation remains notably deficient at the partner level. This phenomenon suggests that females face hidden barriers to promotion to the partner position, limiting their opportunities for upward mobility in their auditing careers. Specifically, Downar et al. (2020) examine the glass ceiling effect for females within audit firms using data from Germany. Although the Chinese auditing market differs from Germany in certain aspects, such as the absence of dominant positions held by the Big 4 international audit firms in China and a more competitive landscape among audit firms, the issue remains relevant in the Chinese auditing market. Based on this, the paper proposes the first hypothesis:

*H1*: Female auditors face a glass ceiling in promotion to partner.

### 2.3 Career dilemmas for female partners

In the labor market, females face a glass ceiling, limiting their opportunities for promotion to top-level positions. However, once females break through this glass ceiling and are promoted to the partner level in the auditing market, they may still encounter challenges and difficulties. These challenges reflect the persistent issue of gender inequality in top leadership despite the significant professional accomplishments of females. Research has shown that even when females are promoted to high-level leadership positions, they are often evaluated less favorably than their male counterparts, especially in male-dominated work environments or when assessed by male evaluators (Eagly et al., 1992). The career prospects for female leaders may also be less favorable than those for male leaders, and they are more likely to find themselves on what is commonly referred to as the “glass cliff” (Ryan and Haslam, 2005). This could be due to various reasons. On the one hand, it may be because females are perceived to lack the competence required in leadership positions and are seen as lacking the necessary experience and skills for leadership roles (Metz and Tharenou, 2001), which can negatively impact a company’s performance (Adhikari, 2012). Therefore, females who are promoted to partner may receive lower evaluations. On the other hand, some scholars argue that the career difficulties females face in leadership roles result from gender bias. Dalton et al. (2014) find that in firms with a higher percentage of female partners, female auditors perceived less gender discrimination. Hardies et al. (2021) provide empirical evidence of gender discrimination in audit firms using data from Belgium. Their research discover that the performance standards for female partners are often higher than those for male partners, yet females are still less likely to be assigned to higher-prestige clients after promotion. Therefore, while females promoted to partner may positively impact companies (Jeong and Harrison, 2017), they may still not receive the recognition they deserve due to gender bias or discrimination. In summary, this paper posits that females who are promoted to partner positions in audit firms may still face career challenges. Despite having overcome the glass ceiling, issues related to gender bias and discrimination may still affect their career progression. In audit firms, maintaining client relationships is a principal task for partners. Retaining existing clients and attracting new ones are essential sources of revenue for partners. Therefore, this paper proposes the second hypothesis:

*H2*: Female auditors are more likely to be assigned to high-risk or less important clients after being promoted to partner.

## 3 Research design

### 3.1 Data and sample

Our sample consists of auditors who participated in auditing listed companies during the period 2014–2021. The data on auditor promotions are manually collected from the China Institute of Certified Public Accountants and other relevant websites. Auditor personal characteristics data are available from the Chinese Research Data Services Platform (CNRDS) database. The rest of the data is from the China Stock Market and Accounting Research (CSMAR) database. We use data at the auditor-year level to test whether there is a glass ceiling for females in auditing careers. As shown in Panel A of Table 1, our sample starts with 18,919 auditor-year observations from 2014 to 2021. After removing data from the year of the auditor’s promotion and the missing values generated by calculating the required variables, the paper ends with 14,377 observations. Panel B illustrates the sample selection process for testing the client assignments of female auditors after they are promoted to partner. In this session, we use the initial sample of 45,348 auditor-company-year observations for 2014–2021. Similar to Panel A, we delete the data for the year of the auditor’s promotion and missing values. Finally, we obtain 31,744 observations to examine the career dilemma females face after breaking through the glass ceiling in the auditing market.

**Table 1:** Sample description.

Panel A: Sample selection for female auditors promoted to partner	
Auditor - year observations (2014–2021)	18,919
Less observations in the year of the auditor’s promotion	(1,133)
Less missing values resulting from calculating the required variables	(3,409)
Auditor-year observations used for regression analysis	14,377
Panel B: Sample selection for client assignments after female auditors were promoted to partners	
Auditor–company-year observations (2014–2021)	45,348
Less observations in the year of the auditor’s promotion	(2,344)
Less missing values resulting from calculating the required variables	(11,260)
Auditor-company-year observations used for regression analysis	31,744

### 3.2 Empirical model and control variables

To test whether females face the glass ceiling in the auditing market, this paper estimates the following multivariate regression model:


(1) $$\begin{array}{l} \text{Promotion}=\alpha_{0}+\alpha_{1}\text{Female}+\text{Controls}+\\ \text{ Year}+\text{AuditFirm}+\varepsilon \; \end{array}$$

where the dependent variable, Promotion, indicates 1 if the auditor is promoted to partner and 0 otherwise. *Female* takes 1 for female auditors and 0 for male auditors. If there is a glass ceiling in the audit market, this paper predicts that females are significantly less likely to be promoted to partner than males. Therefore, a negative coefficient on *Female* is predicted in this paper (i.e., $\alpha_{1}$ > 0).

It also controls for relevant variables that may affect the auditor’s promotion to partner in the following terms: Individual characteristics of the auditor, including whether the auditor graduated with a major in accounting (*Major*), whether the auditor has a master’s degree or higher (*Degree*), the auditor’s working years since obtaining the CPA qualification (*Work_Length*), whether the auditor experienced job-hopping (*Job_Hopping*), as well as whether the auditor possesses industry expertise (*PSFee*); Audit firm-level control variables, including whether it is a Big 4 accounting firm (*Big4*) and the audit firm’s market share (*AF_MarShare*); Client-level control variables, including total assets (*AO_Size*), return on net assets (*AO_ROE*), loss or not (*AO_Loss*), growth (*AO_Growth*), sum of accounts receivable and inventory divided by total assets (*AO_Rece_Inve*), book-to-market ratio (*AO_BTM*), whether it is a state-owned enterprise (*AO_SOE*), the average percentage of independent directors (*AO_Ratio*), whether the chairman and general manager are two positions in one (*AO_Dual*), and the average age of listed firms (*AO_ListAge*) of all the clients audited by the auditor during the year. In addition, the paper controls for year fixed effects and audit firm fixed effects.

To examine the client allocation after female is promoted to partner, this paper estimates the following regression model:


(2) $$\begin{array}{l} \text{ClientAssign}=\alpha_{0}+\alpha_{1}\text{Female}+\alpha_{2}\text{Promotion}+\\ \;\alpha_{3}\text{Female\_Pro}+\text{Controls}+\\ \text{ Year}+\text{Industry}+\varepsilon \end{array}$$

where this paper examines the client allocation after female promotion to partner in terms of risk and importance, respectively. *Female_Pro* represents the interaction term between Female and Promotion.

We measure the client’s risk status by using the variables of whether the client is in financial distress (*Fin_Distress*) and whether it is a high-risk client (*High_Risk*). Following Altman (1968), we define *Fin_Distress* as taking 1 if the client company’s Zscore is less than 1.8, 0 otherwise. *High_Risk* takes 1 if the listed company has been involved in litigation in the previous year, or has been issued a modified audit opinion, or has incurred a loss, otherwise 0. For client importance, it is measured by client total assets and audit fees, that is, client total assets as a percentage of the audit firm’s total assets for all clients during the year (*CI_Size*) and client audit fees as a percentage of the audit firm’s total audit fees for all clients during the year (*CI_Fee*). The larger the value of *CI_Size* and *CI_Fee*, the more important the client is to the audit firm.

Equation (2) controls for the following company-level control variables: client’s total assets (*Size*), return on equity (*ROE*), loss status (*Loss*), growth, sum of accounts receivable and inventory divided by total assets (*Rece_Inve*), book-to-market ratio (*BTM*), whether the company is a state-owned enterprise (*SOE*), the percentage of independent directors (*Ratio*), whether the chairman and general manager are two positions in one (*Dual*), the age of the listed company (*ListAge*), and whether the company changes audit firms (*Chg_Firm*). Furthermore, we also control for the auditor’s characteristics, *Major*, *Degree*, *Work_Length*, *Job_Hopping*, *PSFee* and audit firm characteristics, *Big4*. The industry-fixed effects and year-fixed effects are also controlled. All variables are winsorized at 1 and 99%. Detailed definitions of the variables used in this paper are provided in Appendix A.

## 4 Empirical result

### 4.1 Descriptive statistics

Panel A of Table 2 provides descriptive statistics for female promotion partners. As shown in Panel A, the mean value of Promotions is 0.189, indicating that 18.9% of the auditors in the sample are promoted to partners. *Female* has a mean value of 0.388, showing that 38.8% of females are in the sample. The average value of *Major* is 0.456, indicating that 45.6% of the auditors graduated from accounting-related majors. *Degree* has a mean value of 0.092, indicating that only 9.2% of auditors have a master’s degree or higher. The average working experience of auditors is 9.228 years (*Work_Length*), 12.7% of auditors have experienced job-hopping (*Job_Hopping*), and the proportion of auditors with industry specialization accounts for 12.7% (*PSFee*). Panel B presents descriptive statistics for a sample testing the client allocation after female auditors are promoted to partner.

**Table 2:** Descriptive statistics.

Panel A: Descriptive statistics for the glass ceiling for female promotion to partner								
Variable	Obs	Mean	Min	P25	Median	P75	Max	SD
*Promotion*	14,377	0.189	0.000	0.000	0.000	0.000	1.000	0.391
*Female*	14,377	0.388	0.000	0.000	0.000	1.000	1.000	0.487
*Major*	14,377	0.456	0.000	0.000	0.000	1.000	1.000	0.498
*Degree*	14,377	0.092	0.000	0.000	0.000	0.000	1.000	0.289
*Work_Length*	14,377	9.228	0.000	4.000	8.000	13.000	29.000	5.986
*Job_Hopping*	14,377	0.127	0.000	0.000	0.000	0.000	1.000	0.333
*PSFee*	14,377	0.022	0.000	0.000	0.000	0.000	1.000	0.147
*Big4*	14,377	0.053	0.000	0.000	0.000	0.000	1.000	0.224
*AF_MarShare*	14,377	0.066	0.002	0.018	0.051	0.112	0.164	0.050
*AO_Size*	14,377	22.267	17.277	21.467	22.090	22.878	28.543	1.219
*AO_ROE*	14,377	−0.005	−88.087	0.026	0.067	0.108	33.304	1.676
*AO_Loss*	14,377	0.138	0.000	0.000	0.000	0.000	1.000	0.307
*AO_Growth*	14,377	0.320	−2.733	−0.020	0.110	0.270	263.271	3.456
*AO_Rece_Inve*	14,377	0.260	0.000	0.158	0.247	0.342	0.935	0.144
*AO_BTM*	14,377	0.615	0.009	0.444	0.604	0.778	1.559	0.238
*AO_SOE*	14,377	0.091	0.000	0.000	0.000	0.000	1.000	0.260
*AO_Ratio*	14,377	0.379	0.200	0.333	0.375	0.417	0.750	0.049
*AO_Dual*	14,377	0.302	0.000	0.000	0.000	0.500	1.000	0.403
*AO_ListAge*	14,377	2.192	0.000	1.609	2.303	2.884	3.466	0.765
Panel B: Descriptive statistics of female partner client allocation								
Variable	Obs	Mean	Min	P25	Median	P75	Max	SD
*Promotion*	31,744	0.165	0.000	0.000	0.000	0.000	1.000	0.371
*Female*	31,744	0.324	0.000	0.000	0.000	1.000	1.000	0.468
*Size*	31,744	22.327	20.028	21.406	22.146	23.054	26.331	1.284
*ROE*	31,744	0.046	−1.021	0.026	0.066	0.111	0.329	0.165
*Loss*	31,744	0.121	0.000	0.000	0.000	0.000	1.000	0.326
*Growth*	31,744	0.172	−0.592	−0.025	0.104	0.271	2.694	0.424
*Rece_Inve*	31,744	0.264	0.009	0.143	0.247	0.361	0.724	0.160
*BTM*	31,744	0.610	0.106	0.413	0.601	0.799	1.189	0.257
*SOE*	31,744	0.090	0.000	0.000	0.000	0.000	1.000	0.286
*Ratio*	31,744	0.378	0.333	0.333	0.364	0.429	0.571	0.053
*Dual*	31,744	0.294	0.000	0.000	0.000	1.000	1.000	0.455
*ListAge*	31,744	11.097	0.000	4.000	9.000	18.000	31.000	7.814
*Major*	31,744	0.509	0.000	0.000	1.000	1.000	1.000	0.500
*Degree*	31,744	0.117	0.000	0.000	0.000	0.000	1.000	0.321
*Work_Length*	31,744	13.026	0.000	7.000	13.000	19.000	33.000	6.885
*Job_Hopping*	31,744	0.125	0.000	0.000	0.000	0.000	1.000	0.331
*PSFee*	31,744	0.063	0.000	0.000	0.000	0.000	1.000	0.243
*Big4*	31,744	0.056	0.000	0.000	0.000	0.000	1.000	0.230
*Chg_Firm*	31,744	0.086	0.000	0.000	0.000	0.000	1.000	0.281

Table 3 provides the correlation coefficients for the variables between *Promotion* and auditors’ characteristics. There is a significant negative correlation between *Female* and *Promotion* at the 1% level, indicating that female auditors are less likely to be promoted to partner. This provides initial evidence that female faces glass ceilings in auditing career promotion.

**Table 3:** Correlation analysis.

	*Promotion*	*Female*	*Major*	*Degree*	*Work_Length*	*Job_Hopping*	*PSFee*
*Promotion*	1.000						
*Female*	−0.078***	1.000					
*Major*	−0.005	0.015***	1.000				
*Degree*	0.010*	0.035***	−0.054***	1.000			
*Work_Length*	0.147***	−0.112***	0.162***	0.085***	1.000		
*Job_Hopping*	0.150***	−0.053***	0.034***	0.016***	0.088***	1.000	
*PSFee*	0.037***	−0.034***	0.028***	0.039***	0.119***	0.005	1.000

### 4.2 Tests for glass ceiling in female auditors promotion to partner

Table 4 presents the regression results of equation (1), which examines whether females suffer glass ceilings in partner promotions in the audit market. Columns (1) and (2) are regression results using the OLS model without controlling for fixed effects and controlling for year and audit firm fixed effects. The coefficients of *Female* are −0.085 and − 0.083, respectively, and significant at 1% level. It means that females are significantly less likely to be promoted to partner than males. In economic terms, female auditors have a lower probability of being promoted to partner than males by about 44.6% (−0.083/0.189, where 0.189 is the mean value of *Promotion*) to 45% (−0.085/0.189). It can also be found that the coefficient of *Degree* is significantly positive in both columns, indicating that auditors with higher degrees are more likely to be promoted to partner. *Work_Length* is significantly positively correlated with *Promotion*, suggesting that the longer the working length of the auditor, the more likely to be promoted to partner. The coefficients of *Job_Hopping* and *PSFee* are all significantly positive, demonstrating that auditor’s job-hopping experience and industry expertise positively affect their promotion to partner. To verify the robustness of the conclusions, this paper repeats the above regressions using logit model. Columns (3) and (4) show the regression results for the uncontrolled fixed effects as well as controlling for year and audit firm fixed effects, respectively.^1^ The results reveal that the coefficient for *Female* remains significantly negative using logit regression. Therefore, the findings suggest that females are significantly less likely to be promoted to partner in the audit market than males, and that females face a glass ceiling.

**Table 4:** Tests for glass ceiling in audit market.

	OLS		Logit	
	(1)	(2)	(3)	(4)
*Female*	−0.085^***^	−0.083^***^	−0.832^***^	−0.890^***^
	(−15.21)	(−15.41)	(−14.40)	(−14.43)
*Major*	−0.011^*^	0.004	−0.071	0.050
	(−1.94)	(0.62)	(−1.34)	(0.87)
*Degree*	0.073^***^	0.064^***^	0.513^***^	0.501^***^
	(7.13)	(6.45)	(5.96)	(5.35)
*Work_Length*	0.031^***^	0.031^***^	0.238^***^	0.250^***^
	(57.78)	(57.97)	(50.60)	(45.37)
*Job_Hopping*	0.044^***^	0.026^**^	0.275^***^	0.120
	(4.33)	(2.55)	(3.83)	(1.51)
*PSFee*	0.128^***^	0.121^***^	0.808^***^	0.991^***^
	(5.28)	(5.37)	(5.30)	(6.10)
*Big4*	0.111^***^	−0.170^***^	0.966^***^	0.973^***^
	(7.36)	(−6.33)	(9.25)	(3.37)
*AF_MarShare*	−0.631^***^	−0.503	−5.798^***^	−2.948
	(−10.94)	(−1.51)	(−9.96)	(−0.85)
*AO_Size*	0.003	0.003	0.054^*^	0.064^*^
	(1.00)	(0.86)	(1.79)	(1.94)
*AO_ROE*	−0.038^**^	−0.028^*^	−0.271^**^	−0.183
	(−2.34)	(−1.82)	(−2.14)	(−1.40)
*AO_Loss*	0.024^**^	−0.003	0.222^**^	−0.011
	(2.14)	(−0.24)	(2.19)	(−0.10)
*AO_Growth*	0.007	0.001	0.073	0.014
	(1.24)	(0.13)	(1.32)	(0.24)
*AO_Rece_Inve*	−0.000	0.019	0.068	0.183
	(−0.01)	(1.05)	(0.38)	(0.92)
*AO_BTM*	0.034^**^	0.000	0.304^**^	0.062
	(2.38)	(0.01)	(2.23)	(0.40)
*AO_SOE*	−0.023^**^	0.006	−0.193^*^	−0.011
	(−2.15)	(0.55)	(−1.87)	(−0.10)
*AO_Ratio*	−0.038	−0.027	−0.528	−0.195
	(−0.66)	(−0.48)	(−0.97)	(−0.33)
*AO_Dual*	0.030^***^	0.015^**^	0.281^***^	0.162^**^
	(4.35)	(2.21)	(4.24)	(2.27)
*AO_ListAge*	−0.020^***^	−0.020^***^	−0.204^***^	−0.223^***^
	(−4.89)	(−4.91)	(−5.08)	(−5.23)
*_cons*	−0.082	−0.173^**^	−4.548^***^	−4.117^***^
	(−1.20)	(−2.41)	(−7.32)	(−5.58)
*Year*	Yes	Yes	Yes	Yes
*AuditFirm*	Yes	Yes	Yes	Yes
*adj. R^2^/pseudo R^2^*	0.264	0.324	0.285	0.349
*N*	14,377	14,377	14,377	13,288

***, **, and * denote significance at the 1, 5, and 10% level, respectively.

### 4.3 Tests for career dilemmas after female promotion to partner

Table 5 examines the client allocation of female auditors after being promoted to partners. In particular, column (1) tests the results for *Fin_Distress* as the dependent variable. *Female_Pro* is significantly positive, implying that females are more likely to audit financial distressed clients after being promoted to partner than before. Column (2) shows the probability of assigning high-risk clients after females are promoted to partners. As can be seen, the coefficient of *Female_Pro* is positive and significant at the 1% level, supporting the conjecture that females are more likely to be assigned high-risk clients after being promoted to partner. Column (3) and column (4) test whether females are assigned important clients after being promoted to partner. Column (3) shows the findings using *CI_Size* as the dependent variable, and column (4) shows the regression results using *CI_Fee* to measure client importance. In both column examinations, the coefficients of *Female_Pro* are statistically insignificant, suggesting that females do not audit clients that are important to the audit firm after promotion. These results indicate that female auditors are more likely to audit high-risk clients and less likely to audit significant clients after being promoted to partner. Even if females break through the glass ceiling and become part of the partnership, it is still difficult for them to be taken seriously by audit firms. The findings provide empirical evidence that females face career dilemmas after being promoted to partner, and H2 is supported.

**Table 5:** Tests for career dilemmas after female promotion to partner.

	(1)	(2)	(3)	(4)
	*Fin_Distress*	*HighRisk*	*CI_Size*	*CI_Fee*
*Female*	−0.010^**^	−0.013^**^	−0.001	−0.001
	(−2.28)	(−2.32)	(−0.48)	(−0.45)
*Promotion*	0.008	0.003	−0.002	−0.002
	(1.36)	(0.36)	(−0.96)	(−0.94)
*Female_Pro*	0.022^**^	0.033^**^	0.004	0.004
	(1.97)	(2.10)	(0.93)	(0.93)
*Size*	0.072^***^	−0.021^***^	0.000	−0.001
	(35.28)	(−7.40)	(0.09)	(−0.71)
*ROE*	−0.543^***^	−0.295^***^	−0.024^***^	−0.025^***^
	(−28.85)	(−13.54)	(−3.37)	(−3.48)
*Loss*	0.172^***^	0.146^***^	−0.003	−0.002
	(17.31)	(12.75)	(−0.81)	(−0.74)
*Growth*	0.023^***^	0.039^***^	0.001	0.001
	(4.54)	(5.76)	(0.30)	(0.31)
*Rece_Inve*	−0.071^***^	0.000	0.009^*^	0.009^*^
	(−5.00)	(0.01)	(1.75)	(1.72)
*BTM*	0.568^***^	−0.016	−0.001	−0.002
	(56.25)	(−1.18)	(−0.36)	(−0.51)
*SOE*	0.067^***^	−0.027^***^	−0.006^**^	−0.006^**^
	(8.72)	(−2.89)	(−2.21)	(−2.21)
*Ratio*	−0.005	0.014	−0.019	−0.019
	(−0.16)	(0.29)	(−1.50)	(−1.50)
*Dual*	0.007	0.006	−0.007^***^	−0.007^***^
	(1.64)	(1.06)	(−4.36)	(−4.35)
*ListAge*	0.001^***^	0.010^***^	0.001^***^	0.001^***^
	(3.63)	(24.31)	(5.75)	(5.84)
*Major*	−0.003	−0.002	0.003^**^	0.003^**^
	(−0.86)	(−0.38)	(1.97)	(2.01)
*Degree*	−0.014^**^	−0.015^**^	−0.007^***^	−0.006^***^
	(−2.50)	(−2.01)	(−3.03)	(−2.97)
*Work_Length*	−0.000	−0.000	0.000^***^	0.000^***^
	(−1.01)	(−0.76)	(3.17)	(3.15)
*Job_Hopping*	0.000	−0.012	−0.008^***^	−0.008^***^
	(0.08)	(−1.63)	(−3.68)	(−3.70)
*PSFee*	−0.019^**^	−0.018^*^	−0.005	−0.005
	(−2.45)	(−1.73)	(−1.52)	(−1.42)
*Big4*	−0.065^***^	−0.021^*^	0.018^***^	0.020^***^
	(−6.93)	(−1.85)	(4.98)	(5.53)
*Chg_Firm*	0.024^***^	0.058^***^	0.015^***^	0.015^***^
	(3.35)	(6.13)	(5.06)	(5.05)
*_cons*	−1.660^***^	0.657^***^	0.068^***^	0.080^***^
	(−34.31)	(10.12)	(4.12)	(4.89)
*Year*	Yes	Yes	Yes	Yes
*Ind*	Yes	Yes	Yes	Yes
*adj. R^2^*	0.440	0.089	0.013	0.013
*N*	31,744	31,744	31,744	31,744

***, **, and * denote significance at the 1, 5, and 10% level, respectively.

### 4.4 Tests for gender discrimination faced by the female after promotion to partner

Whether females promoted to partner will be assigned to high-risk or relatively less important clients may be due to multiple reasons, including factors such as relatively lower competence or possibly gender discrimination. As Eagly et al. (1992), Dalton et al. (2014), and Hardies et al. (2021) have found in their studies, females are more likely to be undervalued and more likely to be at risk of gender discrimination, particularly in male-dominated work environments. To further confirm that the difference in client allocation after female promotion to partner is related to gender discrimination in audit firms, this study plans to test whether the difference in client allocation demonstrated in Table 5 is more pronounced in firms with relatively low female representation. Suppose females are relatively underrepresented in some audit firms. In that case, those audit firms may be more inclined to uphold traditional work patterns and biases, leading to more significant differences in client assignments.

To test the above conjecture, this paper divides the sample into two groups based on the yearly median of the proportion of female auditors in audit firms. *High_F* takes 1 if the percentage of female auditors in audit firms is higher than the annual median, and 0 otherwise. Table 6 presents the differences in assigning financial distress clients by female partners across subgroups. As can be seen, the coefficient of *Female_Pro* is significantly positive only in the group with a lower percentage of female auditors. This means that in male-dominated audit firms, they are more likely to be assigned to financially distressed clients after being promoted to partner. Table 7 shows a subgroup test for auditing high-risk clients after female promoted to partner. Similar to the results in Table 6, the coefficient of *Female_Pro* is positive and significant at the 5% level in male-dominated audit firms, supporting the hypothesis of gender discrimination. Furthermore, Table 8 presents subgroup tests of assigning important clients to females after they are promoted to partner. In the subgroup test, *Female_Pro* is significantly associated with *CI_Size* in the group with a higher proportion of female auditors, and the between-group coefficients are significantly different at the 5% level. There were consistent results when using *CI_Fee* to measure the importance of the client to the audit firm. These results indicate that females promoted to partner are only valued in audit firms with a high proportion of females and are allowed to audit important clients. It also provides evidence for gender discrimination.

**Table 6:** Group test for female partners auditing financial distress clients.

Dependent variable *= Fin_Distress*		
	(1)	(2)
	*High_F* = 1	*High_F* = 0
*Female*	−0.006	−0.015^***^
	(−0.93)	(−2.77)
*Promotion*	0.006	0.010
	(0.57)	(1.35)
*Female_Pro*	0.015	0.030^*^
	(0.92)	(1.95)
*Controls*	Yes	Yes
*Year*	Yes	Yes
*Ind*	Yes	Yes
adj. *R*^2^	0.458	0.428
*N*	12,850	18,894
	Prob > chi2 = 0.498	

***, **, and * denote significance at the 1, 5, and 10% level, respectively.

**Table 7:** Group test for female partners auditing high-risk clients.

Dependent variable *= High_Risk*		
	(1)	(2)
	*High_F* = 1	*High_F* = 0
*Female*	−0.008	−0.018^**^
	(−0.87)	(−2.31)
*Promotion*	0.011	−0.004
	(0.79)	(−0.36)
*Female_Pro*	0.009	0.054^**^
	(0.38)	(2.52)
*Controls*	Yes	Yes
*Year*	Yes	Yes
*Ind*	Yes	Yes
adj. *R*^2^	0.079	0.097
*N*	12,850	18,894
	Prob > chi2 = 0.155	

***, **, and * denote significance at the 1, 5, and 10% level, respectively.

**Table 8:** Group test for female partners auditing important clients.

	*CI_Size*			*CI_Fee*		
	(1)	(2)	(3)	(4)	(5)	(6)
	Full sample	*High_F* = 1	*High_F* = 0	Full sample	*High_F* = 1	*High_F* = 0
*Female*	−0.001	0.004	−0.006^***^	−0.001	0.004	−0.006^***^
	(−0.48)	(1.55)	(−2.97)	(−0.45)	(1.58)	(−2.97)
*Promotion*	−0.002	−0.005	−0.000	−0.002	−0.005	−0.000
	(−0.96)	(−1.43)	(−0.03)	(−0.94)	(−1.39)	(−0.03)
*Female_Pro*	0.004	0.012^*^	−0.003	0.004	0.012^*^	−0.003
	(0.93)	(1.66)	(−0.58)	(0.93)	(1.65)	(−0.57)
*Controls*	Yes	Yes	Yes	Yes	Yes	Yes
*Year*	Yes	Yes	Yes	Yes	Yes	Yes
*Ind*	Yes	Yes	Yes	Yes	Yes	Yes
*adj. R^2^*	0.013	0.015	0.015	0.013	0.015	0.015
*N*	31,744	12,850	18,894	31,744	12,850	18,894
		Prob > chi2 = 0.088			Prob > chi2 = 0.090	

***, **, and * denote significance at the 1, 5, and 10% level, respectively.

In conclusion, the above results indicate that female auditors still face career dilemmas after promotion to partner, reflected in the higher likelihood of auditing clients in financial distress, high-risk clients, and lower chances of auditing clients that are important to the audit firm. This situation is more pronounced in male-dominated audit firms. Even if females break through the glass ceiling and become part of the partnership, it is still difficult for them to be taken seriously by audit firms.

## 5 Additional tests

### 5.1 Are females valued after promotion to partner?

In the audit reports of Chinese listed companies, there are at least two signature auditors, acting as review partners (*Leader*) and acting engagement auditors. In particular, the review partner coordinates the audit project and takes ultimate responsibility. Since the audit firms converted to the special general partnership form in 2014, the first signing auditor should theoretically be a partner position in the audit firm. Accordingly, there is a significant increase in the likelihood that females will serve as reviewing partners when they are promoted to partner, regardless of gender discrimination. Additionally, as Hardies et al. (2021), if there is gender discrimination in audit firms, it will require females to perform better than males to achieve promotion. That is, female promotion to partner in audit firms with a high proportion of males will exhibit superior ability, and they are more likely to serve as reviewing partners in audit projects if gender discrimination exists.

The results are shown in Table 9. In the full sample test of column (1), the coefficient of *Female_Pro* is significantly positive at the 1% level. In the subgroup tests in columns (2) and (3), the coefficient of *Female_Pro* is significantly positive in the group with a relatively low share of female auditors, validating the conjecture. In conclusion, the results indicate that females promoted to partner in male-dominated audit firms usually exhibit higher competence (Hardies et al., 2021) and provide evidence of gender discrimination in audit firms.

**Table 9:** Test for whether females are valued by audit firms after promotion to partner.

Dependent variable *= Leader*			
	(1)	(2)	(3)
	Full sample	*High_F* = 1	*High_F* = 0
*Female*	−0.089^***^	−0.085^***^	−0.089^***^
	(−19.69)	(−12.06)	(−14.74)
*Promotion*	0.384^***^	0.378^***^	0.389^***^
	(62.36)	(37.07)	(50.69)
*Female_Pro*	0.054^***^	0.023	0.083^***^
	(4.38)	(1.19)	(5.28)
*Controls*	Yes	Yes	Yes
*Year*	Yes	Yes	Yes
*Ind*	Yes	Yes	Yes
*adj. R^2^*	0.531	0.517	0.544
*N*	31,744	12,850	18,894
		Prob > chi2 = 0.015	

***, **, and * denote significance at the 1, 5, and 10% level, respectively.

### 5.2 Investor reaction to female promotion to partner

Besides, the paper further examines the investors’ earnings response to female promotion to partner. The earnings response coefficient captures investors’ perceptions of the factors affecting a company’s earnings situation. If gender discrimination is prevalent in the capital markets, then this paper argues that investors will react negatively to female promotion to partner and the situation is more pronounced in audit firms with a higher proportion of females. This paper constructs the following model to test investors’ earnings response to female auditor promotion to partner:


(3) $$\begin{array}{l} \text{BHAR}=\alpha_{0}+\alpha_{1}\text{UE}+\alpha_{2}\text{Promotion}+\alpha_{3}\text{Promotion\_UE}+\\ \text{ Controls}+\text{Year}+\text{Industry}+\varepsilon \; \end{array}$$

*BHAR* is the cumulative excess return. Considering that the cut-off date for the publication of listed companies’ financial reports in China is April 30 of the following year, this paper adopts the returns for the 12 months from the beginning of May in year t + 1 to the end of April in year t + 2 to calculate *BHAR*. *UE* is the earnings surprise, expressed as the difference between net profits in period t + 1 and net profits in period t divided by the market value of equity. *Promotion_UE* represents the interaction term between *UE* and *Promotion*. We are interested in the coefficient of *Promotion_UE*, if there is gender discrimination against female auditors in the capital market, then this paper predicts that the coefficient of *Promotion_UE* is negative ($\alpha_{1}<0$).

As predicted, the coefficient on *Promotion_UE* is significantly negative. In the subgroup test, the results show that there is a more significant negative response from investors in audit firms with a higher percentage of females. In contrast, in male-dominated audit firms, investors do not respond significantly to the promotion of female auditors. Consistent with the conjecture, gender bias against female auditors is prevalent at the capital market level (Table 10).

**Table 10:** Investor reaction to female promotion to partner.

	(1)	(2)	(3)
	*Female* = 1	*Female* = 1&*High_F* = 1	*Female* = 1&*High_F* = 0
*UE*	0.709^***^	0.719^***^	0.693^***^
	(12.80)	(9.05)	(9.02)
*Promotion*	0.008	0.010	0.007
	(0.66)	(0.57)	(0.40)
*Promotion_UE*	−0.281^***^	−0.482^***^	−0.065
	(−2.74)	(−3.57)	(−0.40)
*Controls*	Yes	Yes	Yes
*Year*	Yes	Yes	Yes
*Ind*	Yes	Yes	Yes
*adj. R^2^*	0.105	0.099	0.113
*N*	10,010	4,714	5,296
		Prob > chi2 = 0.047	

***, **, and * denote significance at the 1, 5, and 10% level, respectively.

## 6 Conclusion

This study delves deeply into the glass ceiling phenomenon that female auditors face in the auditing market and their career challenges after promotion to partner. The underrepresentation of females in leadership roles has been a hot topic of academic interest, particularly in male-dominated public accounting firms, where this issue becomes particularly pronounced. Despite progress in achieving gender equality in various fields, the number of females entering the public accounting firms has become comparable to that of men. However, the problem of underrepresentation of females, especially in leadership roles such as partner positions, remains a serious concern. In a system where “up or out” is the norm, females’ barriers to advancing to high-level leadership positions could lead to a substantial talent drain in public accounting firms. Therefore, conducting in-depth research into the issues females encounter in their career development within the auditing market is necessary.

Using hand-collected data on auditor promotions to partner in the Chinese audit market, this paper empirically examines the glass ceiling effect of female promotion to partner as well as the career development dilemmas after promotion. First and foremost, the research reveals that, in comparison to their male counterparts, female auditors face significantly lower odds of promotion to partner, thus shedding light on the glass ceiling phenomenon in career development. Secondly, this paper further examines the differences in client allocation following the promotion to partner. The results indicate that females, upon becoming partners, are more likely to be assigned to financial distressed clients and high-risk clients, with fewer opportunities to audit important clients. This difference is particularly pronounced in male-dominated audit firms, underscoring the role of gender discrimination in the observed disparities. Furthermore, this research observes that although female partners have significantly increased opportunities to serve as lead partners in audit projects, these opportunities are primarily concentrated in firms with higher proportions of female auditors. In contrast, females continue to face challenges in gaining full recognition in male-dominated audit firms. Finally, this study tests investors’ reactions to females’ promotion to the partner level, revealing that the capital markets are not optimistic about the career progression of female auditors. These research findings underscore that gender equality remains an important and thought-provoking issue within the public accounting firms.

This study provides significant insights into understanding gender inequality within the public accounting firms and inspires further research. Researchers could delve deeper into the promotion mechanisms within audit firms, particularly focusing on gender equality disparities. This approach would uncover the underlying causes of gender inequality issues, enhancing the effectiveness of potential solutions. Furthermore, future research should comprehensively examine the factors influencing females’ promotion to partner positions, including individual traits, family factors, job performance, and other promotion-related elements. An in-depth exploration of these factors will yield a clearer understanding of the challenges female auditors encounter on their path to partnership and provide precise recommendations for future policies and practices. While this study primarily focuses on the Chinese audit market, its conclusions are applicable globally, as gender inequality issues are widespread in various countries and audit markets. Nevertheless, we encourage future researchers to explore institutional differences in partner promotions within public accounting firms in different countries or regions further. This will aid in pinpointing the specific reasons for gender equality disparities in various locales and provide more compelling empirical support for international gender equality initiatives.

There are some limitations in this paper due to data availability. Firstly, due to data constraints, this paper only observes the promotion process of auditors to equity partner positions. In the Chinese auditing market, some audit firms have salaried partner positions, and while these individuals are also referred to as partners, they do not hold equity in the firm. Their roles fall between auditors and equity partners, similar to director positions in other countries. Consequently, this study does not explicitly examine the promotion of these salaried partners, and it cannot ascertain whether female auditors are more likely to be promoted to salaried partner positions. This is an important aspect to consider when assessing the existence of the glass ceiling phenomenon for female auditors. Secondly, the data in this study is sourced from 40 securities-qualified audit firms, which do not represent the entire landscape of audit firms, particularly smaller ones. This implies that the research findings are somewhat limited due to sample selection. Different-sized and structured audit firms may exhibit variations. Therefore, the results of this study may have limitations when generalized to the entire auditing market. Lastly, we utilize data from the Chinese auditing market, and the structure of auditing markets differs across countries. Consequently, the research findings may not necessarily apply to auditing markets in other countries. Different cultures, regulations, and market conditions can lead to different manifestations of gender inequality issues. Therefore, researchers should exercise caution when applying the results of this study to auditing markets in other nations.

Despite these limitations, this research comprehensively explores female promotion to partner in the auditing market. The findings hold practical significance for public accounting firms and regulatory bodies. Public accounting firms should proactively establish transparent and equitable promotion standards, ensuring that gender does not become a barrier in the promotion process. Industry regulatory bodies should also actively promote gender equality and advocate for diversity and inclusion measures in the audit market. These actions could help increase the representation of females in leadership roles and, consequently, eliminate gender discrimination at the partner level in public accounting firms.

## Data availability statement

Publicly available datasets were analyzed in this study. This data can be found at: https://data.csmar.com/.

## Author contributions

HC: Conceptualization, Data curation, Methodology, Writing – original draft. JW: Formal analysis, Investigation, Validation, Writing – review & editing.

## Footnotes

^1^Column (4) has a sample that is inconsistent with the other three columns because in the logit regression, when year fixed effects and audit firm fixed effects are included, a portion of the sample is lost due to covariance problems.
