# Supplementary material for: What’s in store for females after breaking the glass ceiling? Evidence from the Chinese audit market
Source: Front Psychol. 2023 Nov 21;14:1321391. doi: 10.3389/fpsyg.2023.1321391 (PMC10699082; doi:10.3389/fpsyg.2023.1321391)
Supplement: Supplementary file 1 [file Table_1.pdf]

Appendix A. Variable definitions

Variable	Definition
Promotion	1 if the auditor is promoted to partner, 0 otherwise.
Female	1 if the auditor is female, 0 otherwise.
Major	1 if the auditor graduates from accounting related majors, 0 otherwise.
Degree	1 if the auditor graduated with a master's degree or above, 0 otherwise.
Work_Length	Auditor's working years since qualifying as a certified public accountant (CPA).
Job_Hopping	1 if the auditor has experienced job hopping, 0 otherwise.
PSFee	1 if the auditor has industry expertise, 0 otherwise. Industry expertise is calculated by ranking the auditor's total audit fees for the year in the top 5 in the industry.
Big4	1 if the audit firm is one of the Big 4 international audit firms, otherwise 0.
AF_MarShare	Number of audit firm clients in the year divided by the total number of listed companies in the year.
AO_Size	The average of the total assets (in natural logarithms) of all clients audited by the auditor during the year.
AO_ROE	The average of the return on net assets of all clients audited by the auditor during the year.
AO_Loss	The percentage of clients with a loss for all clients audited by the auditor during the year.
AO_Growth	The average of the growth of all clients audited by the auditor during the year.
AO_Rece_Inve	The average of all clients' *Rece_Inve* audited by the auditor during the year. Where *Rece_Inve* represents the sum of accounts receivable and inventory divided by total assets.
AO_BTM	The average of the book-to-market ratio of all clients audited by the auditor during the year.
AO_SOE	The percentage of state-owned enterprises audited by the auditor during the year.
AO_Ratio	The average percentage of independent directors among all clients audited by the auditor during the year.
AO_Dual	The average value of *Dual* across all clients audited by the auditor during the year. Where *Dual* takes 1 if the chairman and CEO of the company are the same person, otherwise 0.
AO_ListAge	The average of the ages (in natural logarithms) of all clients audited by the auditor during the year.
Size	The natural logarithm of total assets.
ROE	Net profit divided by net assets.
Loss	1 if net profit is less than zero, otherwise 0.
Growth	Growth in sales for the period compared to sales in the last period.
Rece_Inve	Sum of accounts receivable and inventories divided by total assets.
BTM	Book-to-market ratio.
SOE	1 if it is a state-owned enterprise, 0 otherwise.
Ratio	Percentage of independent directors on the board.
Dual	1 if the chairman and CEO of the company are the same person, otherwise 0.
ListAge	Number of years since listing.
Chg_Firm	1 if the client company changes its audit firm during the year, 0 otherwise.
Fin_Distress	If the client company's ZScore for the year is less than 1.8, the company is considered to be in financial distress and takes 1 value, otherwise 0.
HighRisk	If the listed company has been involved in litigation in the previous year, or has been issued a modified audit opinion, or has incurred a loss, it takes 1, otherwise 0.

CI_Size	Total assets of client companies as a percentage of total assets of all listed clients of the audit firm.
CI_Fee	Audit fees of client companies as a percentage of total audit fees of all listed clients of the audit firm.
